# Methodological considerations in region of interest definitions for paraspinal muscles in axial MRIs of the lumbar spine

**DOI:** 10.1186/s12891-018-2059-x

**Published:** 2018-05-07

**Authors:** David B. Berry, Jennifer Padwal, Seth Johnson, Callan L. Parra, Samuel R. Ward, Bahar Shahidi

**Affiliations:** 1 Department of Bioengineering, University of California San Diego, La Jolla, CA USA; 2 Department of Medicine, University of California San Diego, La Jolla, CA USA; 3 Department of Orthopaedic Surgery, University of California San Diego, 9500 Gilman Drive (MS 0863), La Jolla, CA 92093 USA; 4 Department of Radiology, University of California San Diego, 9500 Gilman Drive (MS 0863), La Jolla, CA 92093 USA

**Keywords:** Fatty infiltration, MRI, Lumbar spine, Low back pain, Multifidus, Erector spinae

## Abstract

**Background:**

Magnetic Resonance Imaging (MRI) is commonly used to assess the health of the lumbar spine and supporting structures. Studies have suggested that fatty infiltration of the posterior lumbar muscles is important in predicting responses to treatment for low back pain. However, methodological differences exist in defining the region of interest (ROI) of a muscle, which limits the ability to compare data between studies. The purpose of this study was to determine reliability and systematic differences within and between two commonly utilized methodologies for ROI definitions of lumbar paraspinal muscle.

**Methods:**

T2-weighted MRIs of the mid-L4 vertebrae from 37 patients with low back pain who were scheduled for lumbar spine surgery were included from a hospital database. Fatty infiltration for these patients ranged from low to high, based on Kjaer criteria. Two methods were used to define ROI: 1) segmentation of the multifidus and erector spinae based on fascial planes including epimuscular fat, and 2) segmentation of the multifidus and erector spinae based on visible muscle boundaries, which did not include epimuscular fat. Total cross sectional area (tCSA), fat signal fraction (FSF), muscle cross sectional area, and fat cross sectional area were measured. Degree of agreement between raters for each parameter was assessed using intra-class correlation coefficients (ICC) and area fraction of overlapping voxels.

**Results:**

Excellent inter-rater agreement (ICC > 0.75) was observed for all measures for both methods. There was no significant difference between area fraction overlap of ROIs between methods. Method 1 demonstrated a greater tCSA for both the erector spinae (14–15%, *p* < 0.001) and multifidus (4%, *p* < 0.016) but a greater FSF only for the erector spinae (11–13%, p < 0.001).

**Conclusion:**

The two methods of defining lumbar spine muscle ROIs demonstrated excellent inter-rater reliability, although significant differences exist as method 1 showed larger CSA and FSF values compared to method 2. The results of this study confirm the validity of using either method to measure lumbar paraspinal musculature, and that method should be selected based on the primary outcome variables of interest.

## Background

Low back pain (LBP) is a highly prevalent condition, affecting 65–85% of the general population at some point throughout their lifetime [1]. Magnetic resonance imaging (MRI) is a diagnostic tool that is frequently utilized for evaluation of underlying anatomical pathology, as well as to obtain quantitative measures of spinal kinematics, muscle quality, and size, or injuries such as disc herniation, stenosis, or nerve root compression. Recent studies have highlighted the importance of muscle quality (ie. fatty infiltration) and size (cross sectional area or volume) of the lumbar paraspinal musculature in predicting LBP related disability [2, 3], prognosis for recurrence [4–7], and response to exercise [4]. However, these data are confounded by methodological variation across studies, and as such, the interpretation of results are difficult.

**Fig. 1:**
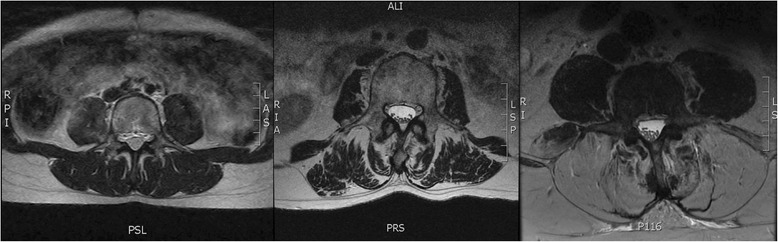
T2 weighted axial MR images of the lumbar spine. Images represent Kjaer grade [16] 0 (left), 1 (center), and 2 (right) muscles of the lumbar spine. All images are from patients undergoing surgery for low back pain related pathology

One important source of variation in MRI-based measures of muscle size and quality is differences between region of interest (ROI) definitions of muscle compartments. Specifically, there is debate about whether or not to include the epimuscular fat “tent” between muscle and the fascial plane in a ROI [8]. Although several studies demonstrate that intra-class correlation coefficient (ICC) values between and within raters is high for a single method [9, 10], reliability and systematic differences across methods have not been established. There is also potential for different methods to result in systematic error in the extremes of the spectrum of muscle quality (i.e. when there are large amounts of fatty infiltrate) due to the differences in methods for determining fascial boundaries between muscles. For example, some ROI definitions may provide high ICC values in cases with low levels of muscle fatty infiltration, but when the muscle has large amounts of fatty infiltrate, the fascial boundaries may become less obvious and potential for error could increase (Fig. 1).

One important consideration related to these methodological differences is that the basis for these ROI definitions could affect interpretation of the underlying physiological processes thought to be occurring with LBP pathologies. Recent studies have supported the idea that pathological changes in muscle are more related to muscle quality, or fatty infiltration, as compared to just muscle size or cross sectional area (CSA) [11, 12]. The underlying biological process related to muscle atrophy is thought to be a result of disuse or decreased metabolic demand [13], which leads to decreases in the size of the muscle compartment. However, fatty infiltration is associated with an accumulation of fat, both within the muscle compartment (intrafascicular fatty infiltration), and outside the epimyseal border (epimuscular fatty infiltration) [14]. These fatty infiltration measures typically quantify fat signal fraction within a defined ROI, and may be largely influenced by the definition of this ROI. Therefore, understanding the magnitude of differences between commonly utilized methods will allow not only for more accurate comparisons of data across studies, but also will allow a more informed interpretation of the underlying physiological changes as a result of pathology. Therefore, the purpose of this study is to determine reliability and systematic differences within and between two commonly utilized methodologies for ROI definitions of the paraspinal muscles in the lumbar spine.

## Methods

### Study participants

MRIs from 37 patients were selected from a larger cohort of 236 patients, who were identified based on current procedural terminology (CPT) codes for lumbar spine surgical procedures between 2005 and 2015 at UC San Diego hospitals. Inclusion criterion for this cohort have been previously described [15]. The patients selected in this study were patients with LBP chosen to represent the full range of muscle fatty infiltrations observed in patients with Kjaer grades 0–2 [16]. All images analyzed in this study were obtained from T2-weighted MRIs at the mid L4 vertebrae to standardize lumbar spine level across patients [17].

### Region of interest definition

**Fig. 2:**
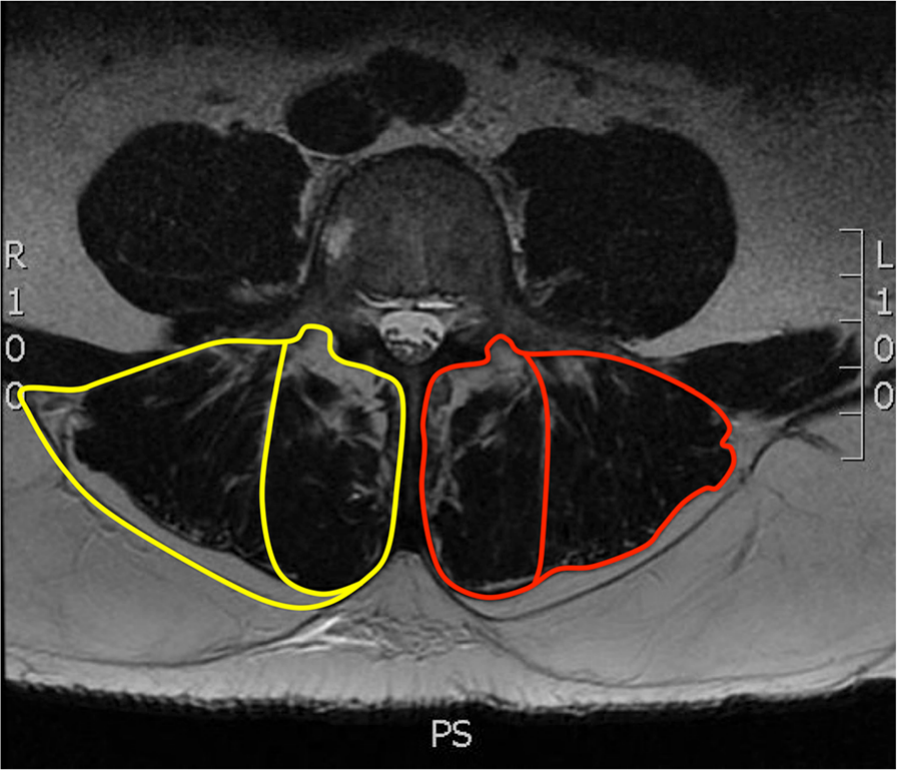
Sample region of interest definitions of the erector spinae and multifidus muscles using Method 1 (yellow) and Method 2 (red)

Regions of interest (ROIs) for both the multifidus and erector spinae muscles were segmented bilaterally using OsiriX software [18]. Two methods of identifying the posterior boundary of the regions of interest were used:

1. Method 1 – Muscle ROI definitions were based on fascial plane separation using the facet joint as a landmark between the multifidus and erector spinae, and the lumbosacral fascia posteriorly. When a large fat-filled “tent” was observed between the longissimus and illiocostalis, this region was *included* in the ROI because it uses the posterior fascial plane as a border (Fig. 2). Additionally, fat tents lateral to the illiocostalis and under the lumbosacral fascial plane were *included* in the region of interest. This technique has previously been defined in Shahidi et al. [15].
2. Method 2 – Segmentation was based on the fascial plane separation using the facet joint as a landmark between the multifidus and erector spinae, and the epimyseal border posteriorly. When a large fat-filled tent was observed between the longissimus and illiocostalis, this region was *excluded* from the ROI (Fig. 2). Additionally, fatty regions lateral to the illiocostalis and under the fascial plane were *excluded* in the region of interest. This technique has previously been defined in detail in Crawford et al. [8].

Three raters with varying levels of experience with lumbar spine muscle ROI measurements (J.P., S.J., B.S.) underwent standardized training based on the strict criteria noted above for both methods. ROI measurements for each method were randomized for each rater and each rater was blinded to prior ROI definitions.

ROIs were then imported into a custom written Matlab software (Mathworks, Natick, MA) to measure total cross sectional area (tCSA), fat signal fraction (FSF), muscle cross sectional area (mCSA), and fat cross sectional area (fCSA). Pixels were identified as either fat or muscle by fitting a two-term Gaussian model to the histogram of pixel intensities from segmented regions of interest, and finding the intersection of the Gaussian distributions. Pixel values above the intersection were classified as fat, and pixels below were classified as muscle. This thresholding method has been previously described in detail [15]. Total cross sectional area was defined as the total area of the ROI for each muscle. tCSA (Eq. 1), FSF (Eq. 2), mCSA (Eq. 3) and fCSA (Eq. 4) were defined as:

1 $$ tCSA=\sum pixel{s}_{fat}+\sum pixel{s}_{muscle} $$

2 $$ FSF=\frac{npixel{s}_{fat}}{npixel{s}_{fat}+ npixel{s}_{muscle}} $$

3 $$ mCSA= tCSA- tCSA\ast FSF $$

4 $$ fCSA= tCSA\ast FSF $$

### Statistical analysis

**Fig. 3:**
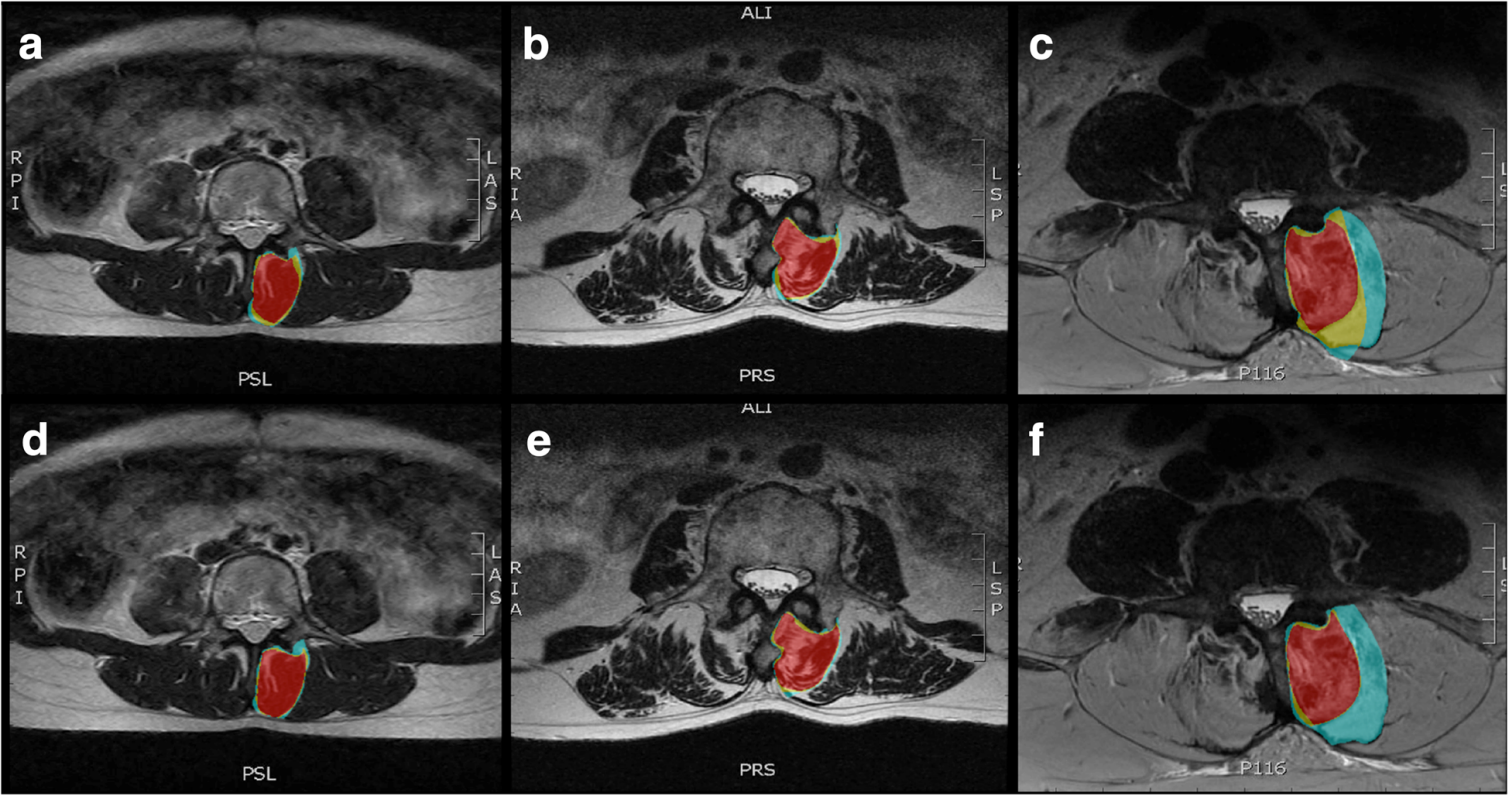
Examples of overlapping regions of interest defined by all 3 raters (red), 2 raters (yellow), or 1 rater (blue) for the multifidus muscle using region of interest definitions from method 1 (**a**-**c**) and method 2 (**d**-**f**) for muscles with Kjaer grade 0 (**a**, **d**), 1 (**b**, **e**), and 2 (**c**, **f**)

**Fig. 4:**
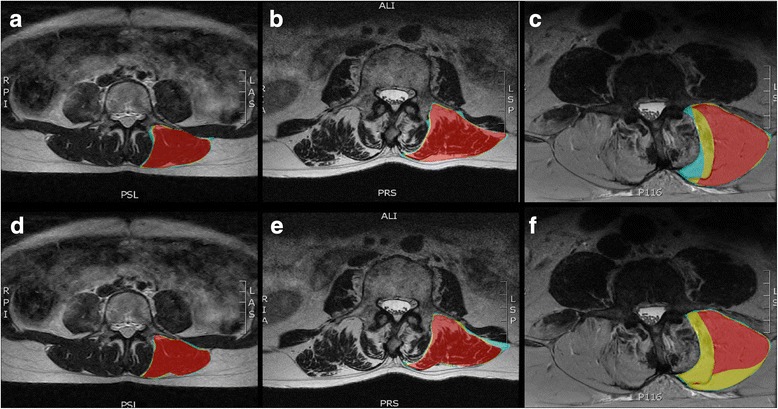
Examples of overlapping regions of interest defined by all 3 raters (red), 2 raters (yellow), or 1 rater (blue) for the erector spinae muscles using region of interest definitions from method 1 (**a**-**c**) and method 2 (**d**-**f**) for muscles with Kjaer grade 0 (**a**, **d**), 1 (**b**, **e**), and 2 (**c**, **f**)

The level of agreement between raters for tCSA, FSF, mCSA, and fCSA was assessed using ICC’s for each muscle and method. ICC estimates and their 95% confidence intervals were based on a mean rating (k = 3), absolute agreement, 2-way mixed effects model. Interpretations of ICC results were based on the guidelines proposed by Cicchetti 1994 [19]: less than 0.40 = poor agreement, 0.40–0.59 = fair agreement, 0.60–0.74 = good agreement, > 0.75 = excellent agreement. In order to assess the how similar the masks drawn by each rater were to each other, area fraction overlap was calculated (Eq. 5), defined as the number of voxels overlapping across all 3 rater masks, divided by the cumulative masked area (Figs. 3 and 4).

5 $$ Area\ fraction\ overlap=\frac{\sum common\ voxels}{total\ area\ of\ voxels\ across\ raters} $$

A paired samples t-test was performed to identify any differences between the overlapping area fractions in the masks (tCSA, FSF, mCSA, and fCSA) of the two methods for each muscle on each side. The relationship between inter-rater coefficients of variation (CV) and absolute tCSA, FSF, mCSA, or fCSA was assessed by linear regression for each muscle and method. All statistics were performed using SPSS Statistics (Version 21, IBM, Armonk, NY). All data are reported as mean ± standard deviation.

## Results

Excellent inter-rater agreement (ICC > 0.75) was observed for all measures for both methods (Table 1). Comparisons between the three raters consistently demonstrated high ICC, with the lowest ICC found for left multifidus tCSA (ICC 0.879; 95% CI 0.761–0.938) and the highest ICC found for right erector spinae FSF (ICC 0.997; 95% CI 0.994–0.998). The ICC’s for method 1 ranged from 0.879 (0.761–0.938) to 0.997 (0.994–0.998) for the left multifidus tCSA and right erector spinae FSF, respectively. The ICC’s for method 2 ranged from 0.928 (0.861–0.963) to 0.995 (0.960–0.997) for the right multifidus tCSA and left erector spinae mCSA, respectively.

**Table 1:** ICC results

Measure	Method	Side	Muscle	ICC	95% confidence interval	
					Lower bound	Upper bound
tCSA	1	Left	Multifidus	0.879	0.761	0.938
			Erector Sp.	0.973	0.951	0.986
		Right	Multifidus	0.886	0.771	0.942
			Erector Sp.	0.971	0.948	0.984
	2	Left	Multifidus	0.946	0.897	0.972
			Erector Sp.	0.979	0.951	0.99
		Right	Multifidus	0.928	0.861	0.963
			Erector Sp.	0.968	0.931	0.984
FSF	1	Left	Multifidus	0.987	0.978	0.993
			Erector Sp.	0.996	0.994	0.998
		Right	Multifidus	0.982	0.969	0.99
			Erector Sp.	0.997	0.994	0.998
	2	Left	Multifidus	0.994	0.99	0.997
			Erector Sp.	0.992	0.976	0.997
		Right	Multifidus	0.980	0.965	0.989
			Erector Sp.	0.991	0.978	0.996
mCSA	1	Left	Multifidus	0.939	0.883	0.968
			Erector Sp.	0.981	0.966	0.99
		Right	Multifidus	0.935	0.882	0.965
			Erector Sp.	0.982	0.969	0.99
	2	Left	Multifidus	0.981	0.961	0.99
			Erector Sp.	0.995	0.990	0.997
		Right	Multifidus	0.972	0.948	0.985
			Erector Sp.	0.994	0.989	0.997
fCSA	1	Left	Multifidus	0.954	0.905	0.977
			Erector Sp.	0.992	0.985	0.995
		Right	Multifidus	0.962	0.905	0.982
			Erector Sp.	0.987	0.975	0.993
	2	Left	Multifidus	0.969	0.945	0.984
			Erector Sp.	0.981	0.944	0.992
		Right	Multifidus	0.953	0.913	0.975
			Erector Sp.	0.963	0.915	0.983

*Erector Sp.* Erector Spinae

The area fraction overlap included in all three ROIs ranged from 0.72–0.85 for method 1 and 0.73–0.83 for method 2. There were no significant differences in the area fraction overlap between methods (*p* = 0.071–0.543). However, a trend was identified for the right erector spinae group, with method 2 having less overlap than method 1 (− 0.05; *p* = 0.071).

There were systematic differences in tCSA, FSF, mCSA, and fCSA between method 1 and method 2 (Table 2). As expected, tCSA was 14–15% larger in the erector spinae (*p* < 0.001) and 4% larger in the multifidus (*p* < 0.016) in method 1 than method 2 because of the inclusion of the lumbosacral fascial border in the ROI definition (Table 2). The inclusion of the posterior fat tent in method 1 also resulted in a 11–13% increase in the overall fat signal fraction for the erector spinae (p < 0.001). Additionally, method 1 measures of erector spinae mCSA and fCSA increased by 5 and 30% respectively (*p* < 0.011). Of note is that while a 6–8% increase in mCSA was measured in the multifidus with method 1 (*p* < 0.037), no increase in fCSA was found (*p* > 0.603).

**Table 2:** Average tCSA, FSF, mCSA, and fCSA measured using both methods

Side	Muscle	tCSA (mm^2^)			FSF		
		Method 1	Method 2	*p*-value	Method 1	Method 2	*p*-value
Left	Erector Sp.	18.54 ± 4.97	16.17 ± 4.06	< 0.001	0.42 ± 0.14	0.38 ± 0.14	< 0.001
	Multifidus	9.08 ± 2.59	8.73 ± 2.44	0.016	0.47 ± 0.18	0.48 ± 0.18	0.216
Right	Erector Sp.	17.88 ± 5.31	15.71 ± 4.23	< 0.001	0.43 ± 0.16	0.38 ± 0.16	< 0.001
	Multifidus	9.14 ± 2.77	8.77 ± 2.48	0.003	0.46 ± 0.18	0.48 ± 0.17	0.209
Side	Muscle	mCSA (mm^2^)			fCSA (mm^2^)		
		Method 1	Method 2	*p*-value	Method 1	Method 2	*p*-value
Left	Erector Sp.	10.63 ± 3.80	10.09 ± 3.65	0.001	7.92 ± 4.08	6.08 ± 2.98	< 0.001
	Multifidus	4.90 ± 2.29	4.62 ± 2.18	0.037	4.18 ± 1.87	4.11 ± 1.71	0.603
Right	Erector Sp.	10.25 ± 4.24	9.85 ± 4.07	0.011	7.62 ± 3.54	5.86 ± 2.88	< 0.001
	Multifidus	4.91 ± 2.32	4.55 ± 2.03	0.011	4.23 ± 2.15	4.22 ± 1.99	0.907

*Erector Sp.* Erector Spinae

**Fig. 5:**
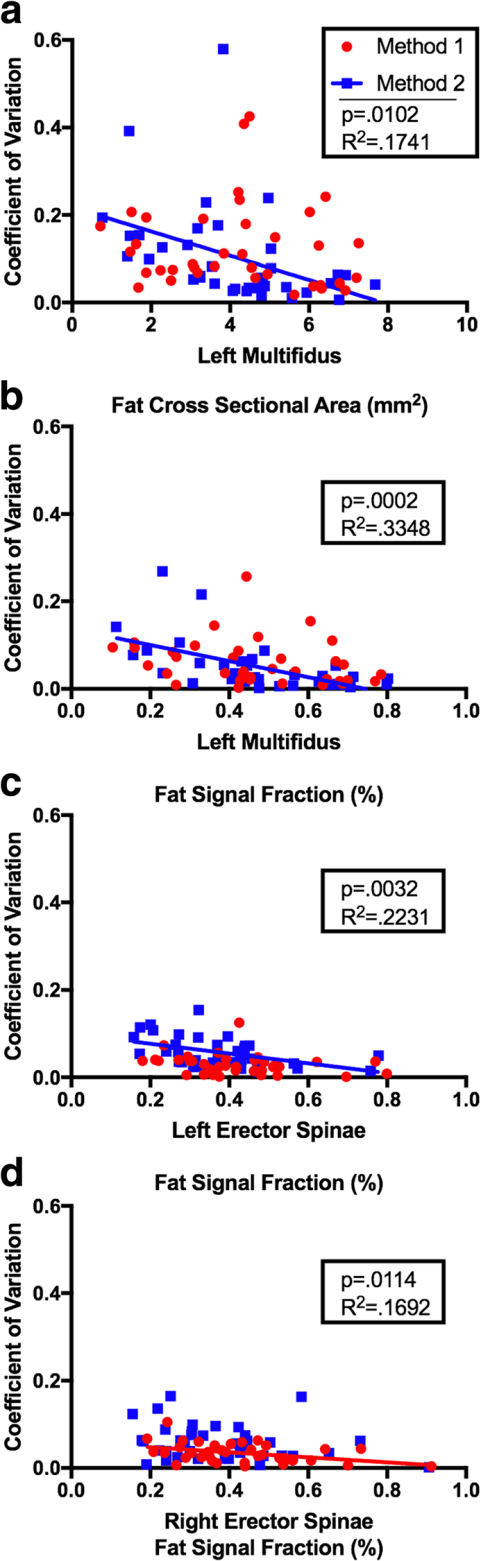
Between-rater error (Coefficient of Variation) decreased with increased left multifidus fCSA (**a**), left multifidus FSF (**b**), and left erector spinae FSF (**c**), for method 2 (blue). Between-rater error decreased with increased right erector spinae FSF (**d**) for method 1 (red)

Overall CV between raters was similar using method 1 (0.08 ± 0.10, range: 0.001–0.77) and method 2 (0.08 ± 0.08, range: 0.001–0.62) for all measures for all muscles. Between-rater error decreased with increased fCSA (*p* = 0.0102, R^2^ = 0.17) and FSF (*p* = 0.0002, R^2^ = 0.33) for in the left multifidus and increased FSF (*p* = 0.0032, R^2^ = 0.22) in the left erector spinae for method 2 (Fig. 5). Additionally, between-rater error was found to decrease with increased FSF (*p* = 0.0114, R^2^ = 0.17) for method 1 only in the right erector spinae muscle, with no other significant relationships between error and outcomes.

## Discussion

This study determined that the reliability for two commonly utilized ROI methods for measuring paraspinal muscle in the lumbar region is high, however there are systematic differences in cross sectional area and fat fraction between the two methods. Method 1, not surprisingly, demonstrates larger CSA and FSF values as compared to method 2. This discrepancy is due to the inclusion of the fatty region between the lumbosacral fascia and the epimysium of the multifidus and erector spinae muscles in method 1, which is excluded in method 2. For both methods, muscle and side specific errors in FSF and fCSA between raters were found to decrease as level of fatty infiltrate increased. This may be due to inherent FSF asymmetry that exists in most patients, which is likely reflected in the anatomical structure and myofascial borders of the muscles of the patients included in this study. Finally, no discrepancy in whole ROI definition (tCSA) was observed between sides using either method.

Muscle volume is a primary input variable for measuring physiological cross sectional area of muscle [20], which is correlated to muscle force generating capacity [20–22]. Muscle CSA is often measured as it is related to muscle volume, and it is relatively easy to measure a single slice across a muscle as compared to the entire volume. As the area fraction of functional contractile tissue decreases, it follows that whole muscle force generating capacity declines, which may result in decreased overall functional capacity. As the erector spinae and multifidus muscles undergo atrophy, the CSA of individual muscle fibers and fascicles decreases, and fat accumulates between the perimysial layers (fascicle atrophy) and epimysium and lumbosacral fascia as a result. In pathological muscle, this atrophy is often accompanied by intrafascicular fatty infiltration, which involves interdigitation of adipocytes throughout the muscle within the perimysial and epimyseal borders [14, 16].

**Fig. 6:**
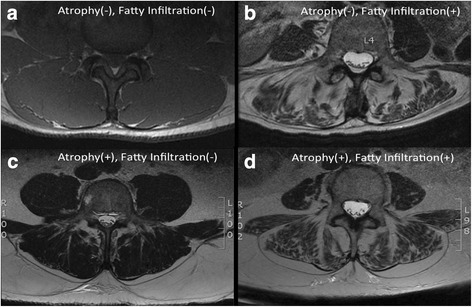
Example MRI axial cross-sections illustrating differences in presentation of muscle quality and size across patients. Panel **a** illustrates a healthy patient with no atrophy or fatty infiltration, demonstrating approximation of the epimyseum and border of the lumbosacral fascia. Panel **b** demonstrates an individual with no epimuscular fatty infiltration, but significant fatty deposition within the muscle compartment. Panel **c** represents a patient with very little muscle fatty infiltration within the epimyseal border, and epimuscular fat accumulation between the lumbosacral fascia and the paraspinal muscle compartments. Panel **d** demonstrates an individual with both intramuscular and epimuscular fatty infiltration

As such, determination of the appropriate method for defining ROI’s in lumbar paraspinal musculature should be dependent on the desired feature of muscle anatomy and physiology to be measured. Prior literature supporting the exclusion of the fatty region in-between the lumbosacral fascia and the epimysium uses the rationale that calculations of muscle area and fatty infiltration should only include the region of tissue within the epimysial border [8]. This definition is likely to provide different information about atrophy versus intrafascicular fatty infiltration. For example, in the normal healthy lumbar spine, the paraspinal epimyseal borders approximate the lumbosacral fascia, and some of the fibers of the multifidus even originate in the lumbosacral fascia [23–25]. As the apparent area between the lumbosacral facial plane and epimyseal border is infiltrated by fat (increased epimuscular fatty infiltration), muscle tCSA would decrease, without a concurrent increase in FSF when measured by method 2 (Fig. 6). Therefore, this method can provide an accurate measurement of intramuscular fatty infiltration in the absence of epimuscular fatty changes. This may be helpful in determining muscle quality within the epimyseal borders, and still yields an accurate measure of mCSA.

In contrast, the inclusion of the epimuscular fat compartment in method 1 would reflect an overall increase in fatty infiltration (both intra- and epi-muscular), which may be a more accurate representation of atrophy for a given individual, based on the observations that in normal healthy muscle (in the absence of atrophy), the epimyseal border and the lumbosacral fascia should approximate each other. However, it would not necessarily provide information distinguishing intramuscular and epimuscular fat. It is unknown currently whether there are biological and functional differences between epimuscular and intramuscular fatty infiltration processes, however, literature suggests that muscle quality, not size, is a more relevant predictor of muscle pathology in individuals with LBP [5, 12, 15]. Importantly, weight does not seem to have an influence on fat fraction when including the epimuscular fat, further suggesting that this is a feature that is independently related to muscle health [15]. Future research is needed to determine whether there are distinct biological processes that differentiate the functional consequences of epimuscular versus intramuscular fatty infiltration.

## Conclusions

In this study, excellent agreement was found between two common methods used to define the regions of interest of the multifidus and erector spinae muscle from axial MRIs. Inclusion of the fat in-between the epimyseal border and the fascial plane results in larger values for tCSA, FSF, fCSA and mCSA when compared to excluding the area of fat, with no differences in variance. The decision to include or exclude the fat area from a region of interest measurement of the lumbar muscles should be made based on the primary outcome a researcher is interested in measuring. Inclusion of the fat area results in a more gross measure of fatty accumulation as a result of atrophy, whereas exclusion of the fatty area may be a more specific measure of muscle tissue quality and possibly degenerative changes within the epimyseal border. Future research is needed to explore the biological mechanisms and functional implications of epimuscular and intramuscular fatty infiltration.

## Abbreviations

CPT: Current procedural terminology
CV: Coefficient of variation
fCSA: Fat cross-sectional area
FSF: Fat signal fraction
ICC: Intra-class correlation coefficients
LBP: Low back pain
mCSA: Muscle cross-sectional area
MRI: Magnetic resonance imaging
ROI: Region of interest
tCSA: Total cross-sectional area
